# Anti-Fibrotic and Anti-Inflammatory Role of NO-Sensitive Guanylyl Cyclase in Murine Lung

**DOI:** 10.3390/ijms241411661

**Published:** 2023-07-19

**Authors:** Nils Englert, Philipp Burkard, Annemarie Aue, Andreas Rosenwald, Bernhard Nieswandt, Andreas Friebe

**Affiliations:** 1 Physiologisches Institut, Julius-Maximilians-Universität Würzburg, 97070 Würzburg, Germany; nils.englert@web.de (N.E.); aue.annemarie@web.de (A.A.); 2 Institute of Experimental Biomedicine, Chair of Experimental Biomedicine I, University Hospital Würzburg, 97080 Würzburg, Germany; burkard_p@ukw.de (P.B.); bernhard.nieswandt@virchow.uni-wuerzburg.de (B.N.); 3 Rudolf Virchow Center for Integrative and Translational Bioimaging, Julius-Maximilians-Universität Würzburg, 97070 Würzburg, Germany; 4 Klinik und Poliklinik für Anästhesiologie, Intensivmedizin, Notfallmedizin und Schmerztherapie, Universitätsklinikum Würzburg, 97080 Würzburg, Germany; 5 Institut für Pathologie, Julius-Maximilians-Universität Würzburg, 97080 Würzburg, Germany; rosenwald@uni-wuerzburg.de

**Keywords:** guanylyl cyclase, inflammation, TGFβ, lung, fibrosis

## Abstract

Pulmonary fibrosis is a chronic and progressive disease with limited therapeutic options. Nitric oxide (NO) is suggested to reduce the progression of pulmonary fibrosis via NO-sensitive guanylyl cyclase (NO-GC). The exact effects of NO-GC during pulmonary fibrosis are still elusive. Here, we used a NO-GC knockout mouse (GCKO) and examined fibrosis and inflammation after bleomycin treatment. Compared to wildtype (WT), GCKO mice showed an increased fibrotic reaction, as myofibroblast occurrence (*p* = 0.0007), collagen content (*p* = 0.0006), and mortality (*p* = 0.0009) were significantly increased. After fibrosis induction, lymphocyte accumulations were observed in the lungs of GCKO but not in WT littermates. In addition, the total number of immune cells, specifically lymphocytes (*p* = <0.0001) and neutrophils (*p* = 0.0047), were significantly higher in the bronchoalveolar lavage fluid (BALF) of GCKO animals compared to WT, indicating an increased inflammatory response in the absence of NO-GC. The pronounced fibrotic response in GCKO mice was paralleled by significantly increased levels of transforming growth factor β (TGFβ) in BALF (*p* = 0.0207), which correlated with the total number of immune cells. Taken together, our data show the effect of NO-GC deletion in the pathology of lung fibrosis and the effect on immune cells in BALF. In summary, our results show that NO-GC has anti-inflammatory and anti-fibrotic properties in the murine lung, very likely by attenuating TGFβ-mediated effects.

## 1. Introduction

Idiopathic pulmonary fibrosis (IPF) is accompanied by alveolar epithelial injury, the formation of α smooth muscle actin (αSMA)-positive myofibroblasts, the production of an extracellular matrix (e.g., collagen), and dysregulated inflammation, leading to functionally impaired lung tissue [1,2]. So far, the underlying mechanisms are not fully understood. Due to the poor prognosis and limited therapeutic options, further studies are required to develop new therapeutic approaches [3,4].

The pro-fibrotic cytokine transforming growth factor β (TGFβ) plays a crucial role in the development of lung fibrosis by mediating the formation of αSMA-expressing myofibroblasts and the production of the extracellular matrix. TGFβ is present in a latent form and must first be activated by various mechanisms [5,6,7]. Cells of the innate immune system, such as macrophages and neutrophils, promote fibrosis via the secretion and/or activation of TGFβ. However, innate immune cells have also been described to exert anti-fibrotic effects [8,9]. The role of the adaptive immune system during lung fibrosis is also controversially debated. Several subtypes of CD4-positive T helper lymphocytes have been identified to trigger milieu-dependent anti- or pro-fibrotic processes [10,11].

Studies with different animal fibrosis models indicate nitric oxide (NO) and its receptor NO-sensitive guanylyl cyclase (NO-GC) to exert anti-fibrotic and anti-inflammatory effects in several organs, including the kidneys, heart, skin, and liver [12,13,14,15]. In bleomycin-challenged triple NO synthase (NOS) knockout mice, fibrotic features such as collagen content and tissue distortion were more pronounced than in the respective controls. In addition, cell differentiation and the determination of interleukins in bronchoalveolar lavage fluid (BALF) revealed a significant increase in lymphocyte and TGFβ levels in triple NOS-KO compared to controls [16]. These results indicate the anti-fibrotic and anti-inflammatory activity of the NO/cGMP pathway in lung fibrosis. In murine lung, we have recently shown that platelet-derived growth factor receptor β (PDGFRβ)-positive pericytes are the major NO-GC-expressing cell type that contributes to a collagen 1-producing subtype of myofibroblasts [17,18]. These results suggest the role of NO-GC in pulmonary fibrosis. However, the exact impact of NO-GC on pulmonary fibrosis remains elusive. The aim of this study was to gain more insight into the antifibrotic role of NO-GC. To evaluate the enzyme’s impact on pulmonary pathology, we subjected NO-GC-deficient mice (GCKO; [19]) to bleomycin-induced lung fibrosis. Our particular focus was the investigation of fibrotic tissue alterations and changes in immune cells in BALF after bleomycin treatment, in combination with TGFβ expression.

## 2. Results

### 2.1. Deletion of NO-GC in Murine Lung

**Figure 1:**
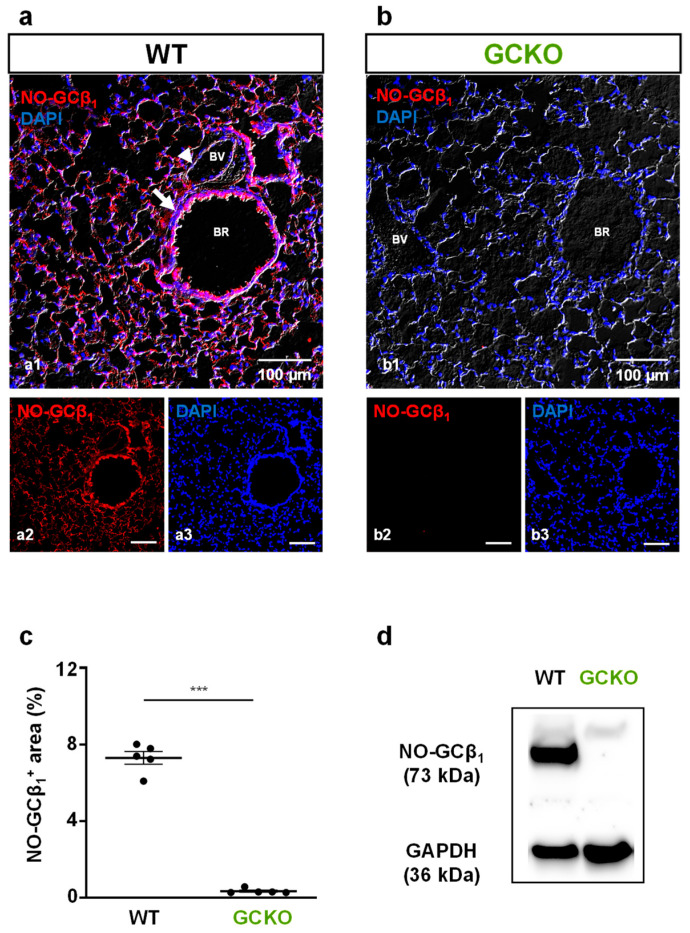
Deletion of NO-GC in murine lung. (**a**,**b**) Murine lung tissues from WT and GCKO were stained with an antibody directed against the β1 subunit of NO-GC (NO-GCβ1; red). (**a**) In WT lung, NO-GC expression was found in the lung parenchyma and in the bronchial and vascular walls. (**b**) In GCKO lung, NO-GCβ1 signals were absent. BR, bronchiole; BV, blood vessel. Single channels are shown in (**a2**,**a3**), (**b2**,**b3**). DAPI was used to stain nuclei (blue). (**c**) Quantitative analysis of NO-GCβ1 signals showed a strong NO-GC expression in WT and verified absence of NO-GC in GCKO lungs (n = 5 animals with 3 images each; Shapiro–Wilk test: not normally distributed). Significance was determined using non-parametric Mann–Whitney test; *** = *p* < 0.001. (**d**) Western blot of lung tissue from WT (n = 4) and GCKO (n = 4) animals showed absence of NO-GCβ1 in the knockout strain. GAPDH is shown as loading control.

We have previously shown that NO-GC is mainly expressed in pericytes and smooth muscle cells in the murine lung [17,18,20]. As the β_1_ subunit of NO-GC (NO-GCβ_1_) is part of each of the enzymatically active NO-GC heterodimers (α_1_/β_1_ and α_2_/β_1_), the use of an NO-GCβ_1_-directed antibody allows quantitative immunofluorescence evaluation of the enzyme. NO-GC is expressed in the lungs of WT mice (Figure 1a) but is not detectable in the lungs of GCKO littermates (Figure 1b). Quantitative analysis of the NO-GC signal corroborated the effective deletion of NO-GC in GCKO lungs (*p* = 0.0007; Figure 1c). Consistent with the immunofluorescence, Western blot analysis showed the absence of NO-GC in lung tissue from GCKO animals (Figure 1d). Taken together, these results clearly show the complete absence of NO-GC in the lung of GCKO mice.

### 2.2. Bleomycin-Induced Lung Fibrosis Is More Pronounced in GCKO

**Figure 2:**
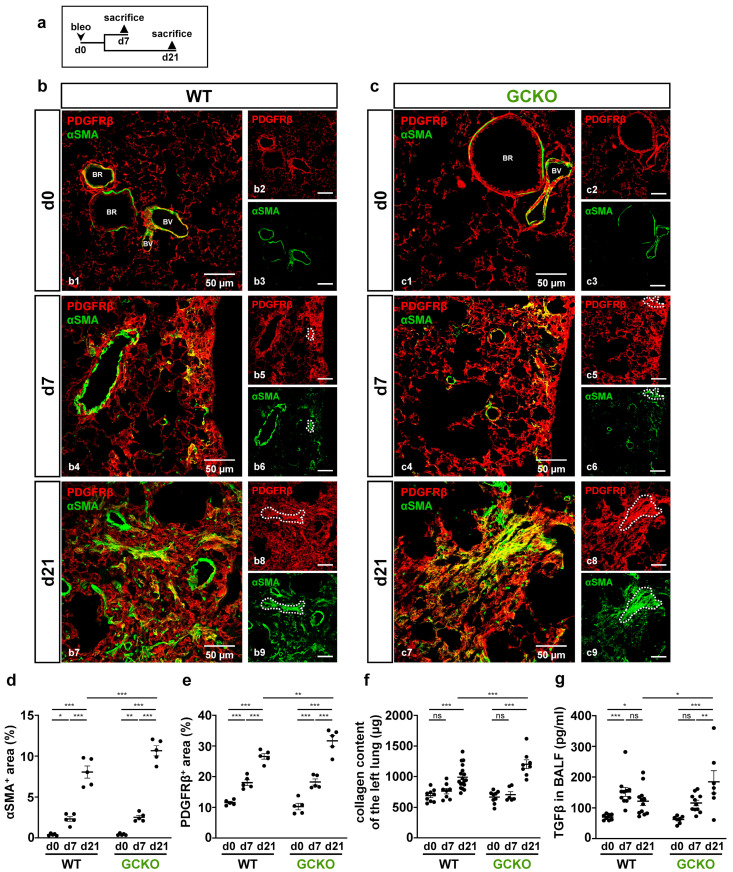
Absence of NO-GC leads to an increased fibrotic response. (**a**) Schematic illustration of the experimental setup of bleomycin-induced lung fibrosis. (**b**,**c**) Lung tissues from untreated (d0) and bleomycin-treated (d7,d21) WT and GCKO animals were stained with antibodies directed against PDGFRβ (red) and αSMA (green). BR, bronchiole; BV, blood vessel. Lung injury was characterized by an increase in PDGFRβ immunosignal and formation of αSMA-positive myofibroblasts in both genotypes (co-expression, yellow; representative areas indicated by dotted lines). Double stainings are shown in (**b1**,**b4**,**b7**) (WT) and (**c1**,**c4**,**c7**) (GCKO); single ingle channels are shown in (**b2**,**b3**,**b5**,**b6**,**b8**,**b9**,**c2**,**c3**,**c5**,**c6**,**c8**,**c9**). Quantitative analyses showed significant increases in PDGFRβ (**d**) and αSMA immunosignals (**e**) in GCKO compared to WT mice after bleomycin treatment (n = 5 animals with 3 images each). (**f**) Hydroxyproline collagen assay displayed a significantly higher collagen content in GCKO compared to WT at day 21 (n = 8–16 animals). (**g**) TGFβ levels in bronchoalveolar lavage fluid (BALF) were measured using ELISA (n = 7–12 animals). Significances were determined using two-way ANOVA followed by Šidák-correction. ns = not significant; * = *p* < 0.05; ** = *p* < 0.01; *** = *p* < 0.001.

To investigate the role of NO-GC in pulmonary fibrosis, WT and GCKO animals were treated with a single orotracheal instillation of bleomycin (2 U/kg); lungs were harvested either 7 or 21 days later (Figure 2a). To compare fibrotic changes, the lung tissues of untreated and treated animals were isolated and then stained with antibodies directed against PDGFRβ and αSMA. 

In healthy tissue, αSMA signals represent vascular and bronchial smooth muscle cells that co-expressed PDGFRβ; αSMA-positive cells in the lung parenchyma (i.e., myofibroblasts) were not detected (Figure 2(b1,c1)). At day 7, PDGFRβ-positive fibrotic foci with αSMA-expressing myofibroblasts were observed in the lung tissue of both genotypes (Figure 2(b4,c4)). 21 days after bleomycin treatment, lung injury was further advanced compared to that on day 7, as illustrated by more widespread PDGFRβ and αSMA immunosignals (Figure 2(b7,c7)). Intriguingly, myofibroblasts showed strong co-expression of αSMA and PDGFRβ (see single channels in Figure 2(b5,b6,c5,c6,b8,b9,c8,c9); dotted lines). Quantitative analyses not only validated the significant bleomycin-induced increase in PDGFRβ and αSMA immunoreactivity in both genotypes over time but also showed that, in GCKO mice, these parameters markedly exceeded those observed in WT at day 21 (αSMA: *p* = 0.0007; PDGFRβ: *p* = 0.0080; Figure 2d,e). Moreover, treatment with bleomycin resulted in a significant rise in collagen content in both genotypes at day 21 compared to non-treatment (Figure 2f). Again, the response in GCKO was significantly higher than that in WT mice at day 21 (*p* = 0.0006). All animals survived until day 7 (see Supplementary Figure S1), yet the mortality of GCKO mice was significantly higher (52%, n = 15/29) than that of WT animals (12%, n = 3/26) when reaching day 21 (*p* = 0.0009; see Supplementary Figure S1). TGFβ, which promotes the formation of myofibroblasts and collagen deposition [5,6,7], was significantly higher in the BALF of GCKO animals compared to WT at day 21 (*p* = 0.0207; Figure 2g), indicating the impact of NO-GC on TGFβ levels. In summary, our data suggest that NO-GC negatively regulates TGFβ expression/activation-mediated effects such as myofibroblast formation and ECM deposition. Therefore, a lack of NO-GC leads to a stronger fibrotic response, explaining the increased mortality of the GCKO animals.

### 2.3. Bleomycin Treatment Affects NO-GC Expression

**Figure 3:**
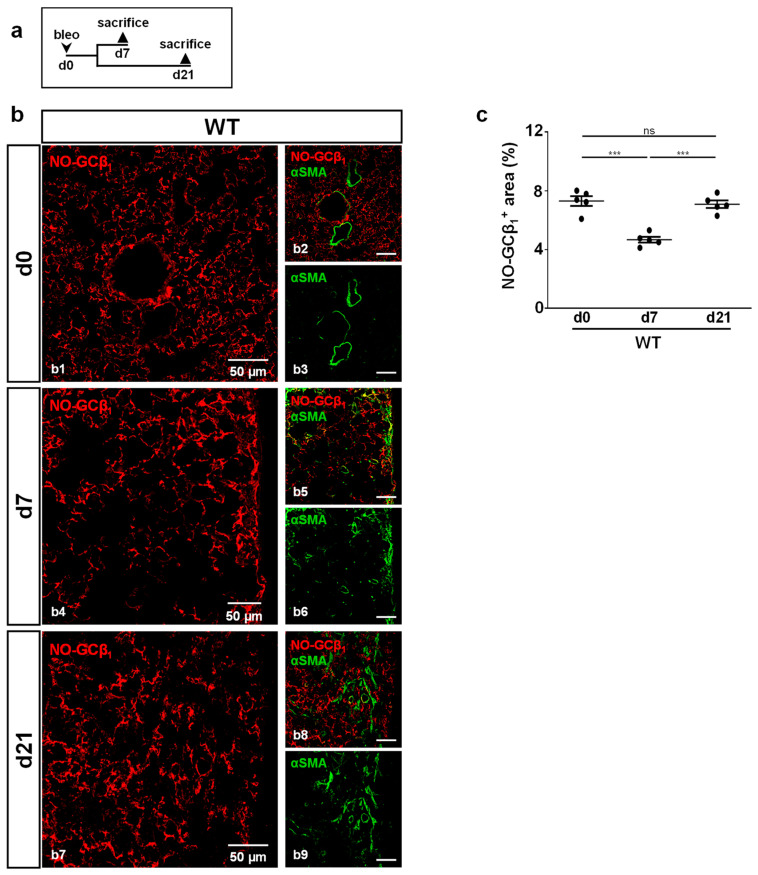
NO-GC expression during murine lung fibrosis. (**a**) Schematic illustration of the experimental setup of bleomycin-induced lung fibrosis. (**b**) Lung tissues from untreated (d0) and bleomycin-treated (d7,d21) WT animals were stained with antibodies directed against the β1-subunit of NO-GC (NO-GCβ1; red) and αSMA (green). Lung injury was characterized by the formation of αSMA-positive myofibroblasts. Double stainings are shown in (**b1,b4,b7**); single channels are shown in (**b2**,**b3**,**b5**,**b6**,**b8**,**b9**). (**c**) Quantitative analyses of NO-GCβ1 signal (αSMA-positive myofibroblasts were used to detect fibrotic regions) represent NO-GC expression in WT lung during bleomycin injury (n = 5 animals with 3 images each; Shapiro–Wilk test: normally distributed). Significances were determined using parametric one-way ANOVA followed by Tukey post-hoc test. ns = not significant; *** = *p* < 0.001.

Next, NO-GC expression was investigated in fibrotic foci (identified by αSMA staining) of WT mice (Figure 3). Mice were treated according to the scheme in Figure 3a. NO-GC was present at any assessed time point (Figure 3b). Quantitative analyses showed a significantly decreased NO-GCβ_1_ signal at day 7 (*p* = <0.0001), followed by a return to original levels at day 21 (*p* = <0.0001; Figure 3c).

### 2.4. Absence of NO-GC Leads to Lymphocyte Aggregation in Fibrotic Lung

**Figure 4:**
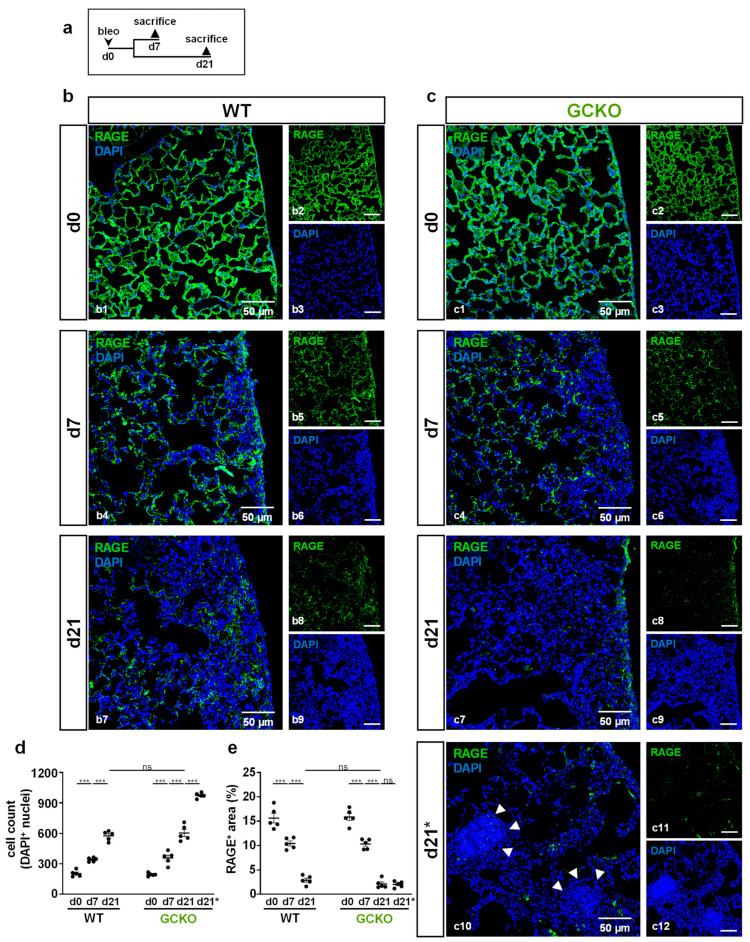
Cell aggregates in bleomycin-treated GCKO lung (**a**) Schematic illustration of the experimental setup of bleomycin-induced lung fibrosis. (**b**,**c**) Lung tissues from untreated (d0) and bleomycin-treated (d7,d21) WT and GCKO animals were stained with an antibody directed against RAGE (green). DAPI (blue) stained cell nuclei. Lung injury was characterized by a reduction of RAGE immunosignal and increase in DAPI-positive cell nuclei. (**c10**) Apart from the injury seen in GCKO tissue at d21 (**c7**) we observed areas of intense DAPI staining in this genotype (indicated by d21*. Cell density in these aggregates (arrowheads) was significantly higher than in regular injured regions at day 21. Double stainings are shown in (**b1**,**b4**,**b7**) (WT) and (**c1**,**c4**,**c7,c10**) (GCKO); ingle channels are shown in (**b2**,**b3**,**b5**,**b6**,**b8**,**b9**,**c2**,**c3**,**c5**,**c6**,**c8**,**c9**,**c11**,**c12**). (**d**) Quantification of DAPI-positive cell nuclei showed a significantly increased cell number in WT and GCKO after bleomycin treatment (n = 5 animals with 3 images each). (**e**) Quantitative analyses of RAGE signal displayed a significant decrease of RAGE immunosignal in WT and GCKO during bleomycin challenge (n = 5 animals with 3 images each). Significances were determined using twoway ANOVA followed by Šidák-correction. ns = not significant; *** = *p* < 0.001.

To investigate bleomycin-induced tissue damage in more detail, histological sections were stained for advanced glycation end products (RAGE) as markers for alveolar epithelial cells type 1 (AEC1; Figure 4). Again, lungs from WT and GCKO animals were studied 7 and 21 days after bleomycin application (Figure 4a).

In untreated animals, AEC1 covered the alveolar surface (Figure 4(b1,c1)). At day 7, injured areas showed a marked reduction in the RAGE immunosignal, indicating destruction of AEC1 (Figure 4(b4,c4),e). In addition, the density of DAPI signals was significantly increased in damaged regions compared to healthy tissue, indicating proliferation/immigration of cells (Figure 4d). In both genotypes, loss of AEC1 and an increase in cell number (evident by reduced RAGE immunosignal and increased number of DAPI-positive nuclei, respectively) progressed until day 21 (Figure 4(b7,c7),d,e). Unexpectedly, lung sections of GCKO mice showed areas with dense accumulations of DAPI-positive nuclei (Figure 4(c10)). These cellular aggregates were only observed in GCKO at day 21 and not in WT. Cell density in these aggregates (=d21*) exceeded that seen in regular injured regions (=d21; *p* = <0.0001; Figure 4d). 

**Figure 5:**
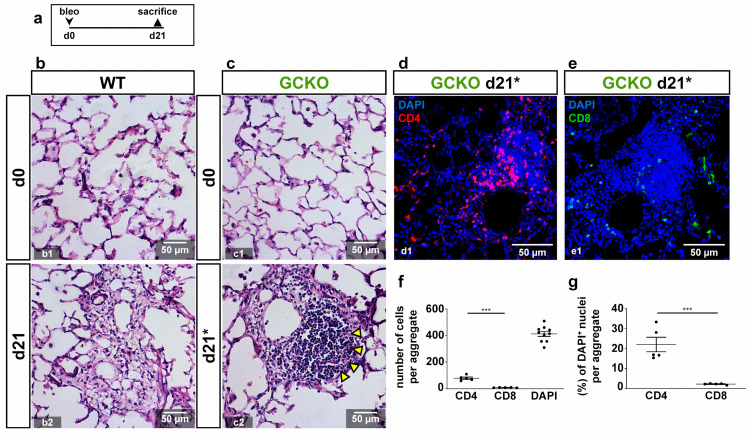
Characterization of lymphocytes in GCKO (**a**) Schematic illustration of the experimental setup of bleomycin-induced lung fibrosis. (**b**,**c**) HE stains from lung tissues from untreated (d0; **b1**,**c1**) and bleomycin-treated (d21; **b2**,**c2**) WT and GCKO animals. Lung injury was characterized by distorted tissue. (**c2**) Arrowheads point to accumulated aggregated lymphocytes in GCKO at d21 (indicated as d21*; see Figure 4(c10)). (**d**,**e**) Lung tissues from bleomycin-treated (d21) GCKO were stained with antibodies directed against CD4 (red) or CD8 (green). DAPI (blue) represented cell nuclei. Lymphocytic aggregates showed relatively strong CD4-positive population (**d**) whereas CD8-positive cells were scarce (**e**). (**f**,**g**) Quantitative analyses of CD4- and CD8-positive cells (n = 5 animals with 3 images each; Shapiro–Wilk test: normally distributed). Significances were determined using parametric unpaired two-tailed *t*-test. *** = *p* < 0.001.

Cell aggregates found in GCKO mice were identified as infiltrated lymphocytes (Figure 5(c2), yellow arrowheads). To further characterize these lymphocytes, CD4/CD8 staining was performed. Izbicki et al. (2002) have already shown that bleomycin treatment increased CD4 lymphocyte numbers (CD4/CD8 T lymphocyte ratio from 1:1 to 2:1; [21]). Immunostaining showed many CD4-positive cells in these lymphocyte aggregates, whereas CD8-positive cells were scarce (Figure 5d,e). Quantification of CD4- and CD8-positive lymphocytes in these aggregates confirmed this observation (absolute: *p* = <0.0001; relative: *p* = 0.0006 Figure 5f,g). As the lymphocyte infiltrations were only observed in GCKO mice, NO-GC might mediate lymphocyte recruitment and/or activity in bleomycin-induced lung fibrosis, thereby exerting anti-inflammatory effects.

### 2.5. Lack of NO-GC Leads to Increased Immigration of Immune Cells in Fibrotic Lung

**Figure 6:**
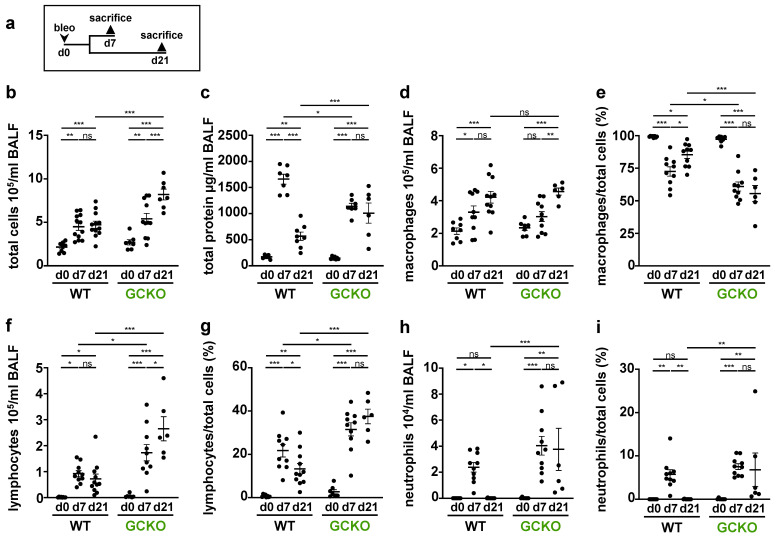
Cell differentiation and total protein levels in BALF (**a**) Schematic illustration of the experimental of bleomycin-induced lung fibrosis. Bronchoalveolar lavage fluid (BALF) from untreated (d0) and bleomycin-treated (d7,d21) WT and GCKO animals was obtained. (**b**,**d**,**f**,**h**) Counting of total immune cells, macrophages, lymphocytes and neutrophils in BALF (n = 7–12 animals). (**c**) Measurement of total protein levels in BALF (n = 6–8 animals). (**e**,**g**,**i**) Percentage of macrophages, lymphocytes and neutrophils (n = 6–11 animals), respectively. Significances were determined using two-way followed by Šidák-correction. ns = not significant; * = *p* < 0.05; ** = *p* < 0.01; *** = *p* < 0.001.

To further investigate the role of NO-GC in immune cell recruitments into the fibrotic lung, WT and GCKO mice were treated as shown in Figure 6a and, subsequently, BALF was obtained for cell differentiation and the determination of protein levels (Figure 6b–i). 

Bleomycin treatment led to an increase in total cell count and total protein concentration in both genotypes. Both parameters were significantly higher in GCKO mice than in WT animals at day 21 (total cell count: *p* = <0.0001; total protein: *p* =0.0003; Figure 6b,c). Interestingly, the protein concentration of WT BALF was significantly higher than that of GCKO at day 7. Under physiological conditions, macrophages are the predominant immune cell type in BALF. Although macrophage numbers increased after bleomycin application (Figure 6d), the percentage of macrophages was reduced (Figure 6e): Of note, the fraction of macrophages was significantly reduced in GCKO compared to WT at day 7 (*p* = 0.0287) and 21 (*p* = <0.0001) even though the absolute macrophage numbers did not differ between both genotypes. In both WT and GCKO, the lymphocyte number and fraction rose significantly after bleomycin application (Figure 6f,g). The absence of NO-GC not only led to the formation of lymphocyte aggregates (see Figure 5) but also to markedly increased intra-alveolar lymphocyte numbers (d21: *p* = <0.0001)/percentage (d21: *p* = <0.0001) compared to WT. In both genotypes, the number and relative amount of neutrophils significantly rose from day 0 to day 7 (Figure 6h,i). Neutrophils increased at day 7 and then returned to basal levels in the WT, whereas in GCKO neutrophil infiltration increased further between day 7 and 21. Taken together, GCKO mice showed a stronger pulmonary immune cell infiltration, especially lymphocytes, than WT mice. In addition, the resolution of acute inflammation was impaired in GCKO, as indicated by the increased number of neutrophils at day 21 (compared to WT: *p* = 0.0047). Thus, NO-GC exerts an anti-inflammatory function on the bleomycin-challenged lung.

### 2.6. Immune Cell Number Correlates with TGFβ

**Figure 7:**
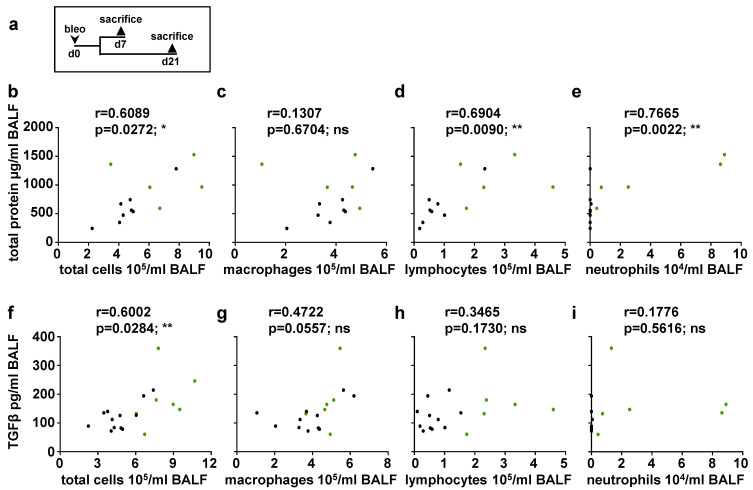
Correlation analyses between total protein/TGFβ and number of immune cells at day 21 (**a**) Schematic illustration of the experimental setup of bleomycin-induced lung fibrosis. Correlation analyses of bleomycin-treated (d21) animals were performed. (**b**–**e**) Correlations between total protein levels and number of total immune cells, macrophages, lymphocytes or neutrophils were determined (n = 13 animals; green dots indicate GCKO, black dots WT). (**f**–**i**) Correlations between TGFβ levels and number of total cells, macrophages, lymphocytes or neutrophils were determined (n = 17–19 animals; green dots indicate GCKO, black dots WT). Correlations were determined using Pearson correlation. r = Pearson correlation coefficient. ns = not significant; * = *p* < 0.05; ** = *p* < 0.01.

Next, we analyzed a possible correlation between the total protein/TGFβ levels and the number of immune cells (Figure 7). At day 0 and 7, no correlation could be observed (see Supplementary Figures S2 and S3). However, at day 21, a positive correlation was observed between total protein levels and either the total number of immune cells, lymphocytes, or neutrophils (Figure 7b,d,e), but not regarding the number of macrophages (Figure 7c). The causal relationship between immune cell increased and intra-alveolar protein accumulation needs to be clarified in further studies. TGFβ concentration and total number of immune cells showed a positive correlation (Figure 7f), whereas TGFβ content could not be associated with an individual immune cell type at day 21 (Figure 7g–i). It is tempting to speculate that all immune cells contribute significantly to the pro-fibrotic environment by activation/secretion of TGFβ [22,23,24] and other protein factors. Thus, our data indicate that not a single immune cell population but rather the inflammatory cells collectively mediate pro-fibrotic changes in bleomycin-induced lung fibrosis.

## 3. Discussion

### 3.1. Deletion of NO-GC in Murine Lung

Recently, we identified smooth muscle cells and pericytes as the major NO-GC-expressing cell types in the murine lung [17,18]. However, the role of NO-GC during pulmonary fibrosis is still unknown. Using immunofluorescence and Western blot analyses, we here verified the absence of NO-GC in murine lung in the NO-GC knockout strain (GCKO; [19]). Under control conditions, mice lacking NO-GC showed fatal gastrointestinal obstruction and hypertension [19]. When subjected to the bleomycin-induced lung fibrosis model, GCKO mice displayed increased pulmonary fibrosis and inflammation compared to WT, showing that NO-GC mediates anti-fibrotic and anti-inflammatory effects in murine lung fibrosis. The relevance of the GCKO phenotype can be assumed but needs to be clarified in detail in further studies.

### 3.2. Interaction of TGFβ and NO-GC

**Figure 8:**
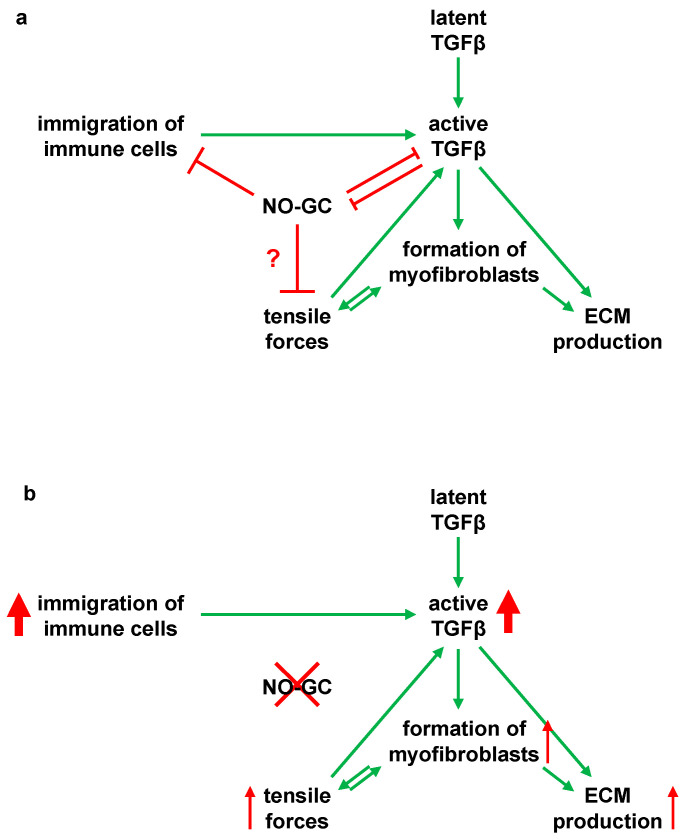
Model for an anti-inflammatory and anti-fibrotic role of NO-GC in murine lung fibrosis (**a**) Schematic illustration of the possible interaction of NO-GC with TGF-β and immune cell immigration in bleomycin-induced pulmonary fibrosis. Moderate wound healing is present, as found in WT mice. In WT lungs, NO-GC regulates the immigration of inflammatory cells, which contribute to the release and/or activation of TGF-β. NO-GC and TGF-β inhibit each other and are at equilibrium. Following activation of TGFβ by proteases, integrins or tensile forces, the pro-fibrotic cytokine promotes differentiation of αSMA-positive myofibroblasts as well as ECM synthesis. By forming a functional syncytium, the myofibroblasts generate shear forces via the αSMA fibers. This traction activates latent TGF-β and also stimulates myofibroblast differentiation. NO-GC may regulate these tensile forces by mediating relaxation of αSMA fibres. There is a balance between the NO-GC and TGFβ-mediated effects as well as immigration of immune cells. (**b**) Schematic representation of the interactions in NO-GC-deficient mice (GCKO). The absence of NO-GC negatively influences the regulation of immune cell immigration and inhibition of TGFβ-mediated effects. This leads to an increased inflammatory reaction, which results in excessive fibrotic activity via an increased TGFβ concentration.

TGFβ mediates pro-fibrotic effects such as the promotion of αSMA expression, differentiation of myofibroblasts, and ECM deposition [5,6,7]. The anti-fibrotic effects of NO-GC in pulmonary fibrosis have already been postulated, as in vitro and in vivo findings indicate NO-GC to suppress TGFβ-mediated effects [25,26,27,28]. Here, we show that a lack of NO-GC leads to significantly higher TGFβ levels in bleomycin-challenged murine lungs compared to the respective control at day 21. Elevated TGFβ levels were paralleled by an increased myofibroblast occurrence, evident by enhanced αSMA and de novo PDGFRβ expression as well as collagen deposition in GCKO compared to the WT control. These findings strongly point to the anti-fibrotic effect of NO-GC by attenuating TGFβ activation/expression and, thus, TGFβ-mediated effects (see Figure 8; possible interactions based on existing literature). The underlying mechanisms and responsible cells are still not known. However, pericytes may be involved, based on a recent study that describes the protective effect of NO-GC-expressing pericytes in acute lung injury [29]. In summary, NO-GC is required for physiological wound healing and the absence of NO-GC leads to excessive fibrotic activity in bleomycin-induced pulmonary fibrosis. 

In addition, TGFβ is also known to inhibit NO-GC: In vitro and in vivo data have revealed that TGFβ signaling downregulates the expression of the α_1_ subunit of NO-GC in murine lung [30]. We have recently shown that the number of NO-GC-expressing cells increases but that these cells, at the same time, reduce their NO-GCβ_1_ mRNA expression during lung fibrosis [18]. Although the exact mechanism is unknown, our in vivo data indicate that TGFβ and NO-GC regulate each other in WT mice: At d7, after bleomycin application, the TGFβ concentration was increased (see Figure 2g), while the protein level of the NO-GCβ_1_ was reduced (see Figure 3c); at d21, the levels of both proteins reversed to almost basal levels. This reversal appears to be mediated by NO-GC since, in the absence of the enzyme, TGFβ levels at d21 did not reverse but were increased even further (see Figure 2g). Thus, it is conceivable that the TGFβ signaling and NO-GC signaling pathways negatively regulate each other during murine lung fibrosis (see Figure 8; possible interactions based on the existing literature).

### 3.3. Anti-Inflammatory Role of NO-GC in Pulmonary Fibrosis

NO-GC is thought to regulate the invasion of immune cells [31]. In this study, we show that lymphocytes, as well as other immune cells, are recruited into fibrotic foci after bleomycin challenge. This process was stronger in GCKO lungs compared to WT lungs. In addition, lymphocyte aggregates were only found in lungs of GCKO mice. These findings suggest the role of NO-GC in immune cell immigration and, thus, in the inflammation process during lung fibrosis. Further research is required to identify the relevant cell type(s) and underlying mechanisms. However, the involvement of pericytes and/or platelets (both of which express high levels of NO-GC; [20]) is conceivable. 

The impact of immune cells on bleomycin-induced lung fibrosis is still controversially discussed [8,9,10,11]. Although our data do not show causality, we assume that the pro-fibrotic impact of immune cells is mediated by TGFβ. TGFβ expression cannot be assigned to a specific immune cell population but our results indicate the joint action of the entire subset of inflammatory cells (macrophages, neutrophils, and lymphocytes). Of course, other cell types, such as alveolar epithelial cells type 2 or fibroblasts, may be involved in TGFβ secretion and activation.

The mice used in this study lack NO-GC globally. Although the lung phenotype of our GCKO mice is obvious, we are aware that this phenotype may be dependent on individual cell types in the lung and influenced by other cell types in the body. The identification of NO-GC-expressing cells directly influencing TGFβ and lymphocyte accumulation, i.e., the use of cell/promoter-specific animals, is therefore mandatory. Further limitations result from the exact mechanism by which the NO-GC and TGFβ signaling pathways crosstalk. Similarly, the NO-GC dependence of mediators that influence lymphocyte accumulation must be studied in the future.

In conclusion, our results indicate the role of NO-GC on immune cell behavior and TGFβ activation/secretion in bleomycin-induced lung fibrosis (see Figure 8). The absence of NO-GC leads to increased immune cell immigration and to higher TGFβ levels, which may jointly result in increased fibrosis. Consequently, NO-GC is very likely involved in anti-inflammatory and anti-fibrotic reactions in murine pulmonary fibrosis by a mechanism that has yet to be analyzed.

## 4. Materials and Methods

### 4.1. Animals

Mice lacking NO-GC (GCKO; genetic background: C57Bl6) were generated as described previously [19]. WT littermates were used as controls. Animals were housed in standard mouse cages (267 × 207 × 140 mm; maximally three animals/cage) with woodchip bedding material and under conventional laboratory conditions (constant room temperature (22 °C), humidity level (55%), and a 12-h light/12-h dark cycle (lights on at 6 a.m.). Since GCKO animals survive better when fed a fiber-reduced diet supplemented with omeprazole/bicarbonate (Altromin, Lage, Germany), WT controls received the same diet for better comparison. Water was available ad libitum. 

### 4.2. Bleomycin Administration 

Lung fibrosis was induced by a single orotracheal administration of bleomycin sulfate (in 0.9% sodium chloride; 2 U/kg body weight). Then, 7 or 21 days later, lungs were harvested. Initial tests showed no difference between the untreated control mice and control mice receiving the same volume of 0.9% sodium chloride solution; therefore, untreated (d0) mice served as controls.

### 4.3. Bronchoalveolar Lavage and Cell Differentiation

After euthanasia, the trachea was exposed and cannulated using a 20-gauge venous catheter. Lungs were rinsed three times with 600 µL ice-cold PBS and the obtained BALF was collected in a 1.5 mL tube. Samples were centrifuged at 400× *g* for 10 min at 4 °C. The supernatants were transferred to safe lock 1.5 mL tubes, immediately snap frozen in liquid nitrogen, and stored at −80 °C. The cell pellets were resuspended in 500 µL ice-cold 2% (*w*/*v*) bovine serum albumin (BSA) in PBS. Samples were centrifuged at 400× *g* for 10 min at 4 °C, supernatants were discarded, and pellets were resuspended with 100 µL Ammonium-Chloride-Potassium (ACK) lysis buffer. Subsequently, 500 µL 1x PBS was added. A mixture of 10 µL sample and 10 µL trypan blue solution was pipetted into a Neubauer improved counting chamber, and total cell counts were determined in a blinded fashion. Using a cytospin centrifuge (Shandon Cytospin 4, Thermo Scientific, Dreieich, Germany; 800 rpm, 10 min), cytospin preparations of 5 × 10^4^ cells were obtained. Samples were air-dried for 2 h at room temperature and stained using a DiffQuick staining kit (HAEMA—LT-SYS^®^ Labor + Technik Eberhard Lehmann GmbH, Berlin, Germany). Object carriers were mounted with Eukitt^®^ cover medium. Images were captured with a light microscope (Thunder Imager, Leica, Wetzlar, Germany). At least 500 cells were differentiated manually in a blinded fashion using ImageJ. Proportions of different cell types were extrapolated to the total cell count of the respective sample.

### 4.4. BCA Assay

To determine total protein levels in the BALF, a BCA assay was performed in a blinded fashion. The snap frozen cell-free BALF was thawed and protein concentrations were determined using BCA assay (Pierce™ BCA Protein Assay Kit, Thermo Scientific). Absorption was measured spectrophotometrically at 562 nm using a microplate-reader (Spark 10 M, Tecan, Männedorf, Switzerland).

### 4.5. Quantification of Interleukins

Protein concentration of TGFβ in BALF was analyzed by ELISA (Mouse TGF-beta 1 DuoSet ELISA; DY1679; R&D Systems, Minneapolis, MN, USA). The snap frozen supernatants of BALF were thawed and ELISA was carried out according to the manufacturer’s instructions. The undiluted samples were activated using a sample activation kit (DY010; R&D Systems, Minneapolis, MN, USA). Duplicates of standards and samples were transferred to a 96-well plate and the protein concentration was determined spectrophotometrically at 450 nm using a microplate-reader (Spark 10 M, Tecan).

### 4.6. Immunofluorescence Analysis

After the animals were sacrificed, lungs were perfused with 0.9% sodium chloride solution and 4% paraformaldehyde (PFA; in 1× PBS) through the right ventricle. Using a 20-gauge needle through a small incision into the trachea, lungs were inflated to 24 cm H_2_O pressure with 4% PFA. Inflated lungs were removed and fixed with 4% PFA for 20 min. The tissue was incubated overnight in 20% sucrose (in PBS) and subsequently snap frozen. For immunofluorescence, 10 µm cryosections were cut, air-dried, permeabilized with 0.1% Triton X-100, and incubated overnight in a water vapor-saturated atmosphere with the following primary antibodies: homemade antibody against the β_1_-subunit of NO-GC (NO-GCβ1; ~360 N-terminal amino acids fused to glutathione-S-transferase; e.g., in [19,32]) raised in rabbit (1:800), goat anti-PDGFRβ antibody (AF1042; 1:200; R&D Systems, Minneapolis, MN, USA), mouse anti-αSMA FITC-conjugated antibody (F3777; 1:500; Sigma-Aldrich, München, Germany), rat anti-receptor for advanced glycation end-products (RAGE) antibody (ABIN360934; 1:100; Antibodies-online Inc., Atlanta, GA, USA), rat anti-CD4 antibody (MCA1767T; 1:500; Bio-Rad Laboratories Inc., Hercules, CA, USA) and rat anti-CD8 antibody (MCA609GT; 1:500; Bio-Rad Laboratories Inc., Hercules, CA, USA). Secondary antibodies were incubated in antibody diluent either alone or in combination for one hour: The rabbit antibody was detected by a donkey anti-rabbit Alexa 555-IgG antibody (A-31572; 1:500; Invitrogen, Darmstadt, Germany), the rat antibodies were detected by a donkey anti-rat Alexa-647-conjugated IgG antibody (A78947; 1:500; Invitrogen, Darmstadt, Germany), and the goat antibody was detected by a donkey anti-goat Alexa 647-conjugated IgG antibody (A-21447; 1:500; Invitrogen, Darmstadt, Germany). Sections were stained with DAPI (A4099; 1:1000; Applichem, Heidelberg, Germany) for 7 min and, finally, mounted with Mowiol. Images were captured using a confocal microscope (TCS SP8, Leica). To capture HE-stained sections (3 µm), a light microscope (Axio Imager 2, Zeiss, Oberkochen, Germany) was used.

### 4.7. Quantification of 20× Immunofluorescence Images

Representative 20× images from different animals (n = 5 animals with 3 images each; averaged value from 3 images was given for each animal) were captured under identical settings. Using macro scripts for Fiji, areas of αSMA, PDGFRβ, RAGE, and NO-GC immunostaining were determined. One quarter (290 µm × 290 µm) of a whole 20× image (580 µm × 580 µm) was analyzed. Regions of interest (ROI) were chosen without SMC of blood vessels and bronchi. DAPI-positive cell nuclei as well as nuclei which were surrounded by CD4- or CD8-immunosignal were counted using macro scripts for Fiji.

### 4.8. Western Blot

Mice were sacrificed by cervical dislocation in isoflurane anesthesia. Lungs were rinsed three times with 0.9% sodium chloride solution and isolated. Tissue was then homogenized with a glass/glass potter (5× volume homogenization buffer) and homogenates were centrifuged (1000× *g*, 4 °C, 3 min). Supernatant was used for Western blot analysis. Protein concentration was determined using Bradford assay. Separation of the cytosolic proteins (20 µg of lung homogenate per lane) was conducted by gel electrophoresis. Western blot was performed using antibodies against the β_1_-subunit of NO-GC (NO-GCβ1; 1:500; homemade; specificity shown, e.g., [19]) and GAPDH (2118; 1:1000; Cell Signaling, Frankfurt, Germany). 

### 4.9. Hydroxyproline Assay

After euthanasia, the left pulmonary lobe was excised and dried to measure lung dry weight. The left lung was hydrolyzed in 6 M HCl (100 µL per 1 mg) for 18 h at 115 °C and centrifuged for 2 min at 9500× g. The supernatants were dried in a Speed-Vac centrifuge (Concentrator Plus, Eppendorf, Enfield, CT, USA) and used for measurements of collagen in a hydroxyproline assay. The pellets were resuspended and diluted 1:40 with water. Samples and standards (in triplicates and duplicates, respectively) were oxidized with chloramine T for 20 min at room temperature. Next, Ehrlich’s reagent was added, and the solutions were incubated for 15 min at 60 °C. Absorbance was spectrophotometrically determined at 560 nm (Wallac 1420 Victor2, Perkin Elmer, Waltham, MA, USA), and collagen content was calculated on the basis of the standard curve. The dry lung weights were used for standardization.

### 4.10. Materials

Chloramine T trihydrate, trans-4-Hydroxy-L-proline, 4-(dimethylamino) benzaldehyde and other standard chemicals were purchased from Sigma (Taufkirchen, Germany). 

### 4.11. Statistics

GraphPadPrism 9.0 for Windows was used to calculate the statistical tests. Data are expressed as mean ± SEM. The statistical tests applied are noted at the appropriate place in the figure texts. Data were tested for normal distribution by the Shapiro–Wilk normality test. According to the result of the Shapiro–Wilk test, two independent groups were compared applying the parametric unpaired, two-tailed *t*-test or non-parametric Mann–Whitney test. Parametric one-way ANOVA followed by Tukey post-hoc test was performed to compare multiple, normally distributed groups of one genotype. Two-way analysis of variance (ANOVA) followed by Šidák-correction was used to test two genotypes with multiple groups. Correlation analyses were performed using Pearson correlation.

## Data Availability

The data presented in this study are available on request from the corresponding author.

## Supplementary Materials

The following supporting information can be downloaded at: https://www.mdpi.com/article/10.3390/ijms241411661/s1.
