# Supplementary material for: Anti-Fibrotic and Anti-Inflammatory Role of NO-Sensitive Guanylyl Cyclase in Murine Lung
Source: Int J Mol Sci. 2023 Jul 19;24(14):11661. doi: 10.3390/ijms241411661 (PMC10380760; doi:10.3390/ijms241411661)
Supplement: Supplementary file 1 [file ijms-24-11661-s001.zip › ijms-2466607-supplementary.pdf]

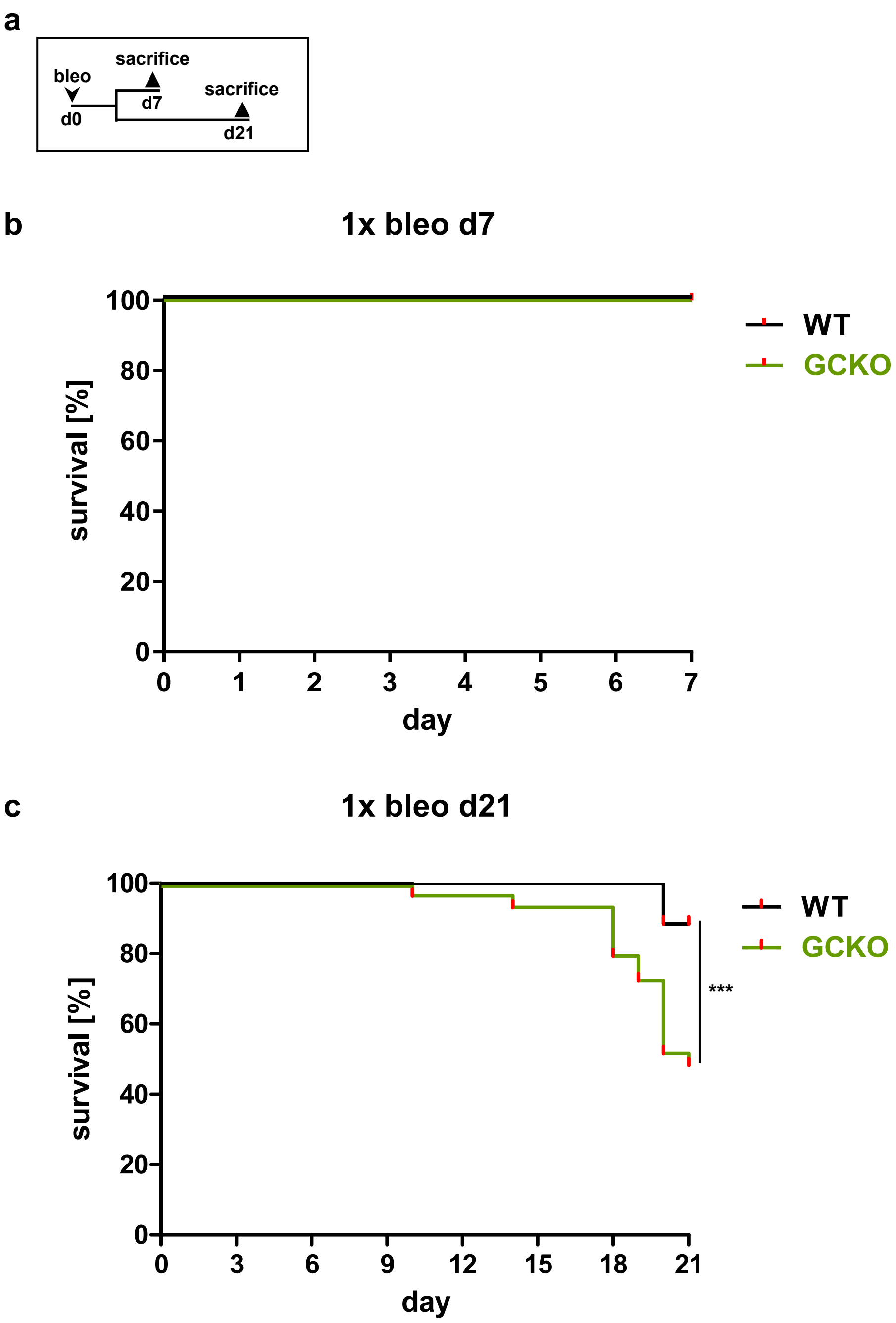

Supplementary Figure S1: Survival after bleomycin treatment.
(**a**) Schematic illustration of the experimental setup of bleomycin-induced lung fibrosis. (**b**) All WT (n=20/20; 100%) and GCKO (n=17/17; 100%) littermates survived to sacrifice (day 7). (**c**) Significantly more WT (n=23/26; 88%) than GCKO animals (n=14/29; 48%) survived to sacrifice (day 21). Note that also here all animals survived at least the first 7 days. Significance was determined using Log-rank (Mantel-Cox) test. ***=p<0.001.

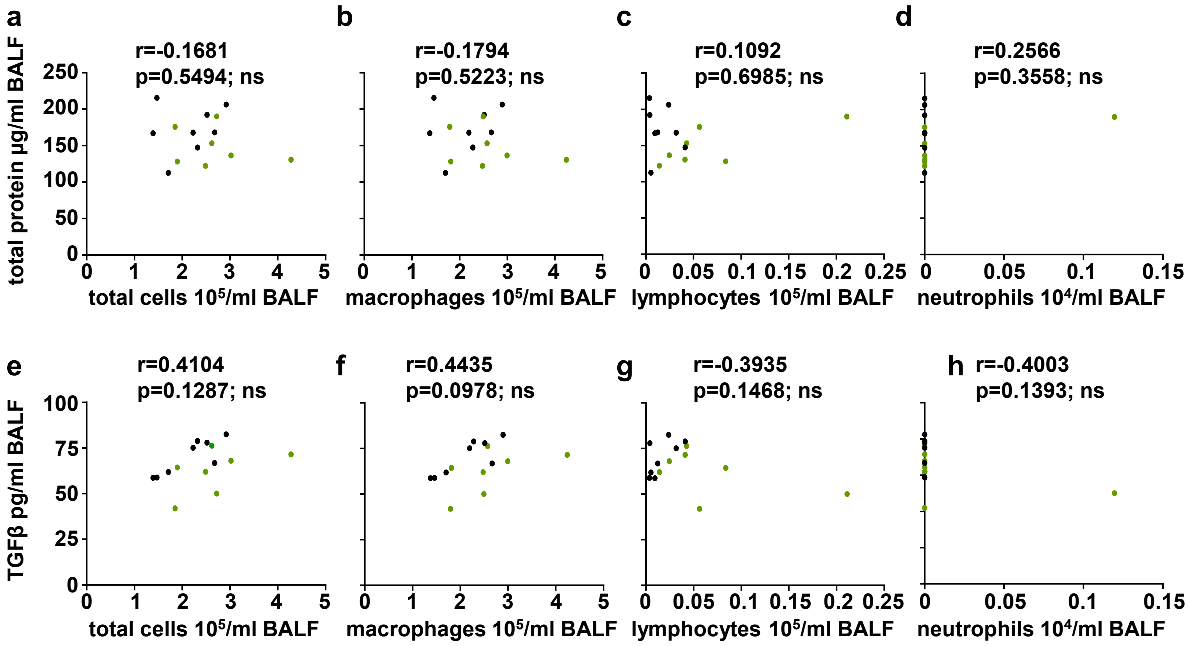

Supplementary Figure S2: Correlation analyses between total protein/TGFβ and number of immune cells at day 0.

Correlation analyses of untreated (d0) animals were performed. (**a-d**) Correlations between total protein levels and number of total immune cells, macrophages, lymphocytes or neutrophils were determined (n=15 animals; green dots indicate GCKO, black dots WT). (**e-h**) Correlations between TGFβ levels and number of total cells, macrophages, lymphocytes or neutrophils were determined (n=15 animals; green dots indicate GCKO, black dots WT). Correlations were determined using Pearson correlation. r=Pearson correlation coefficient. ns= not significant.

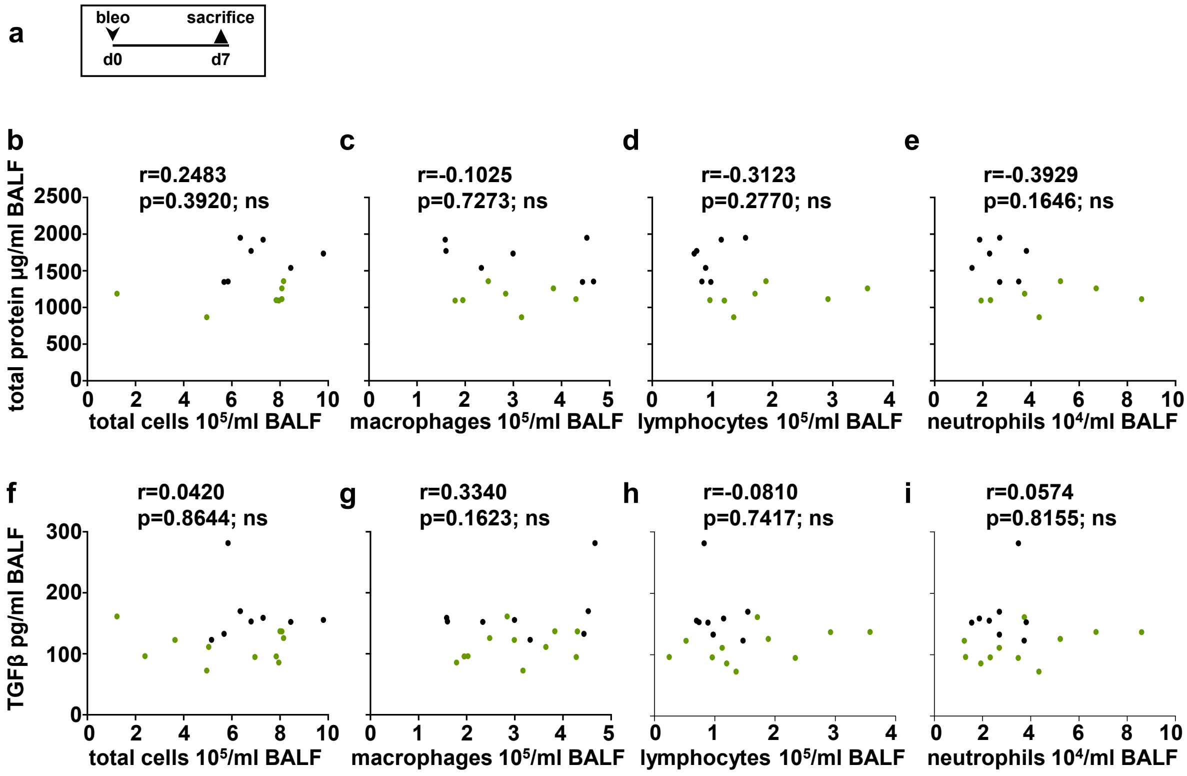

Supplementary Figure S3: Correlation analyses between total protein/TGFβ and number of immune cells at day 7.

(**a**) Schematic illustration of the experimental setup of bleomycin-induced lung fibrosis. Correlation analyses of bleomycin-treated (d7) animals were performed. (**b-e**) Correlations between total protein levels and number of total immune cells, macrophages, lymphocytes or neutrophils were determined (n=14 animals; green dots indicate GCKO, black dots WT). (**f-i**) Correlations between TGFβ levels and number of total cells, macrophages, lymphocytes or neutrophils were determined (n=19 animals; green dots indicate GCKO, black dots WT). Correlations were determined using Pearson correlation. r=Pearson correlation coefficient. ns= not significant.
